# Bacteriophage T5 dUTPase: Combination of Common Enzymatic and Novel Functions

**DOI:** 10.3390/ijms25020892

**Published:** 2024-01-10

**Authors:** Anatoly Glukhov, Victor Marchenkov, Ulyana Dzhus, Antonina Krutilina, Georgii Selikhanov, Azat Gabdulkhakov

**Affiliations:** 1 Institute of Protein Research RAS, 142290 Pushchino, Russia; gluktol@gmail.com (A.G.); march@phys.protres.ru (V.M.); ulya@vega.protres.ru (U.D.); selikhanov@vega.protres.ru (G.S.); 2 International Institute “Solution Chemistry of Advanced Materials and Technologies”, ITMO University, 191002 St. Petersburg, Russia; 3 Almetyevsk State Petroleum Institute, 423450 Almetyevsk, Russia

**Keywords:** bacteriophage, T5, dUTPase, deoxyuridine triphosphate nucleotidohydrolases, dUTP

## Abstract

The main function of dUTPases is to regulate the cellular levels of dUTP and dTTP, thereby playing a crucial role in DNA repair mechanisms. Despite the fact that mutant organisms with obliterated dUTPase enzymatic activity remain viable, it is not possible to completely knock out the *dut* gene due to the lethal consequences of such a mutation for the organism. As a result, it is considered that this class of enzymes performs an additional function that is essential for the organism’s survival. In this study, we provide evidence that the dUTPase of bacteriophage T5 fulfills a supplemental function, in addition to its canonical role. We determined the crystal structure of bacteriophage T5 dUTPase with a resolution of 2.0 Å, and we discovered a distinct short loop consisting of six amino acid residues, representing a unique structural feature specific to the T5-like phages dUTPases. The removal of this element did not affect the overall structure of the homotrimer, but it had significant effects on the development of the phage. Furthermore, it was shown that the enzymatic function and the novel function of the bacteriophage T5 dUTPase are unrelated and independent from each other.

## 1. Introduction

Deoxyuridine triphosphate nucleotidohydrolases (dUTPases, or Duts) are produced by most living organisms as part of their pyrimidine biosynthesis networks [1]. Their role is to eliminate excess dUTP, which can be mistakenly incorporated into DNA by DNA polymerases, since most of them cannot distinguish between dUTP and dTTP. In addition, dUTPases provide the dTTP precursor, dUMP [2]. The indispensability of dUTPase genes has been demonstrated in various organisms, including bacteria [3,4], yeast [5], and mice [6]. Moreover, many viruses and phages also possess the genes for encoding dUTPases.

Based on their oligomerization state, dUTPases can be classified into three families: monomeric [7], homodimeric [8], and homotrimeric [9] enzymes. The homotrimeric family is the largest and includes dUTPases found in various organisms such as plants, animals, fungi, bacteria, and certain viruses like adenoviruses, poxviruses, and retroviruses [10].

It has been described in previous publications that dUTPases can exhibit supplementary functions in addition to their dUTPase activity. For instance, trimeric dUTPases encoded by staphylococcal phages have been shown to induce the transfer of staphylococcal pathogenicity islands (SaPI) by interacting with the SaPI-encoded Stl repressor [11,12,13]. This mechanism relies on the presence of dUTP and is reminiscent of the nucleotide-dependent signaling mechanism seen in eukaryotic G proteins. Interestingly, this function is not related to the dUTPase enzymatic activity itself, but is determined by an extended segment (loop) of the polypeptide chain up to 40 amino acids in length in the protein structure. In contrast, the *Staphylococcus epidermidis* phage PH15 dUTPase, which lacks this loop, is unable to induce SaPI transfer. Another example is the dUTPase of *Mycobacterium smegmatis*, in which the deletion of a mycobacteria-specific loop near the C-terminal region does not significantly affect its dUTPase enzymatic properties, but proves to be lethal for the cell [4]. This suggests the existence of an essential additional function associated with that loop.

Our study focuses on investigating the involvement of the homotrimeric dUTPase, which is synthesized during the bacteriophage T5 infection of *Escherichia coli*, in the process of phage development. In the study by Warner H.B. et al. [14], it was shown that during the T5 phage infection of *E. coli* cells, the overall level of dUTPase activity increased. As a consequence, they obtained a viable mutant phage through random mutagenesis in which this activity was not observed during its development in host cells. This is interesting because viral genomes typically follow the pattern of occupying as little space as possible, suggesting that each gene serves a purpose and is often vital for efficient phage development. This raises the question of why the T5 phage possesses its own dUTPase, if its primary function is not essential for its life cycle. We aim to uncover the additional function performed by T5 dUTPase during phage development that has allowed it to be preserved throughout evolution and to determine which structural element is responsible for this function.

Here, we conducted a comparison of the dUTPases from the T5 phage, T5-like phages, and the host enzyme dUTPase of *E. coli* (Figure S1). Although there is limited sequence similarity between the T5 phage dUTPase (T5Dut) and the *E. coli* dUTPase, the comparison of their spatial structures revealed a high degree of similarity, except for the presence of a specific loop in the T5 and T5-like phages that is absent in the bacterial protein. We presume that this role is facilitated by the structural element unique to the T5 and T5-like phages, which enables the phage dUTPase to carry out an additional function that is not compensated by the presence of the *E. coli* dUTPase.

## 2. Results

### 2.1. Extra Function of T5Dut in Addition to dUTPase Activity

As previously mentioned, there is evidence that the dUTPase of the T5-like phages, besides its primary role in DNA repair, may also play an additional role in the development of phages within the host cells [11,12,13,15]. To more widely investigate this fact, a mutant T5 phage with an amber mutation in the *dut* gene was generated using site-directed mutagenesis. Phage progeny derived from crossing the phage T5 wild-type with plasmid pZ/T5dut-am (Supplementary Materials) were analyzed, and several plaques exhibiting phenotypic differences (the formation of smaller phage plaques) when grown on the *E. coli* XAC (Su0) non-suppressing strain were selected. *Dut* gene sequencing of these phages confirmed the presence of amber mutations. One of these mutant forms was further used and designated as T5*dut*-am. In amber mutant bacteriophage T5*dut*-am development, no dUTPase production was traced. Furthermore, it was observed that the amber mutation in the T5*dut*-am phage genome could be effectively complemented by the wild-type *dut* gene nucleotide sequence when the mutant phage was plated on the *E. coli* XAC strain containing the pBADex1/T5dut plasmid. Conversely, no complementation effect was observed when the mutant (T5*dut*-am) phage was plated on the *E. coli* XAC strain containing the pBADex1/Ecdut plasmid consisting of the *E. coli* dUTPase gene. These findings strongly support the importance of this enzyme in the development of T5 bacteriophages within *E. coli* cells and suggest the existence of an additional function inherent to phage dUTPase.

**Figure 1:**
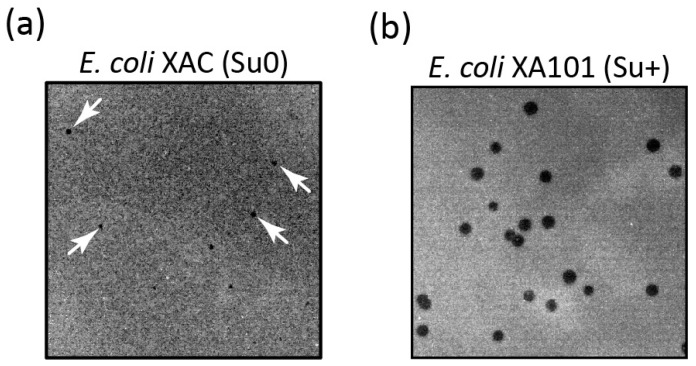
Phenotypic manifestation of an amber mutation in the T5 phage *dut* gene. The mutant phage, T5*dut*-am, was plated with the same dilution onto a lawn of non-suppressing (XAC) (plaques are marked with arrows) (**a**) and suppressing (XA101) (**b**) *E. coli* strains.

It is worth noting that the manifestation of the *dut* gene mutation in the T5 phage exhibits some peculiarities. When the mutant T5 phage is plated on the *E. coli* XAC (Su0) strain, very small and slightly visible colonies are formed (Figure 1a), and their quantity is approximately three times less compared to those obtained by plating them on the *E. coli* XA101 (Su+) (Figure 1b)-suppressing strain (Table 1). Additionally, experiments aimed at determining the burst size of the phage demonstrated that this specific mutation results in a nearly tenfold decrease in phage yield.

### 2.2. The Additional Structural Element of the T5 Phage dUTPase Is Responsible for Carrying Out the Novel Function

**Figure 2:**
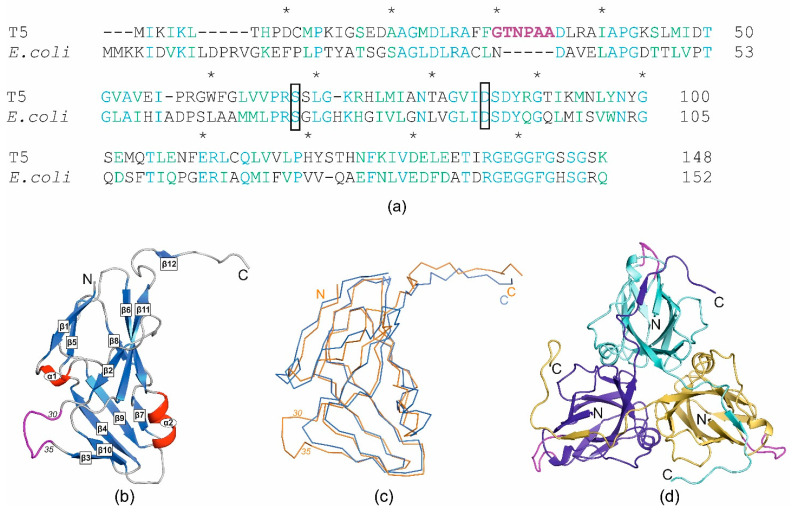
(**a**) Sequence alignment of trimeric dUTPases (identical residues are shown in blue, homologous residues in green, and every tenth residue is marked with an asterisk); the extra loop is shown in magenta, and the mutated residues of the enzymatic active center (Ser68 and Asp85) are boxed; (**b**) schematic ribbon representation of the monomer wild-type T5Dut structure (α-helices in red and β-strands in blue), with the phage-specific insert highlighted in magenta; (**c**) ribbon model of the monomer of T5Dut (orange) superimposed on the *E. coli* dUTPase monomer (blue, pdb id 1DUD); (**d**) trimer of T5Dut structure; the extra loop is shown in magenta.

In the previously observed cases of double dUTPase function, the specific structural elements associated with the supplemental action were identified [12,13]. The comparative analysis of dUTPase sequences from bacteriophage T5 and the host organism *E. coli* revealed a low identity of only 34% (Figure 2a). Additionally, both gaps and insertions were observed, making it difficult to confidently identify the regions presumed responsible for performing the additional function. To assess the significance of these differences in the spatial structures of the given proteins, the crystal structure of T5Dut was obtained at a resolution of 2.0 Å, pdb id 8QKY (Table 2). Despite the low identity, the overall tertiary and quaternary dUTPase structures of the phage and host were found to be very similar, with a root-mean-square deviation (RMSD) of 1.3 Å when aligning the Cα atoms of the monomers. The structure shows the typical trimeric dUTPase antiparallel β-pleated fold of the subunits (Figure 2b). Three subunits are attached to each other in the same manner as observed in other trimeric dUTPases (Figure 2d), forming three active sites per enzyme molecule in the region of monomer interaction.

**Figure 3:**
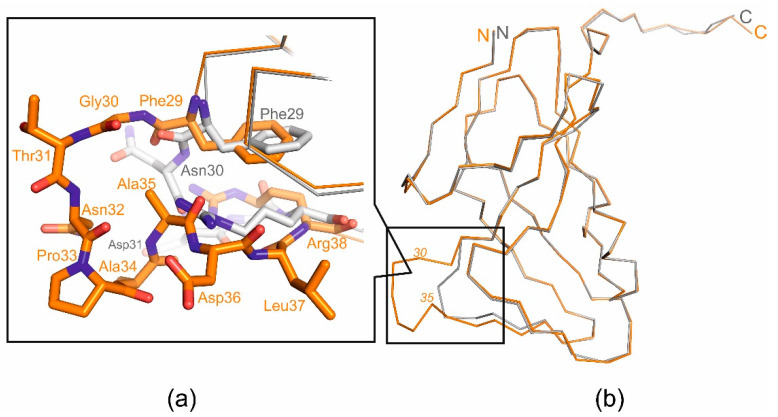
Superimposed structural view of the T5Dut (orange, pdb id 8QKY) and the mutant form T5DutΔloop (gray, pdb id 8QLD): (**a**) close-up of the loop region; (**b**) monomer superposition.

**Figure 4:**
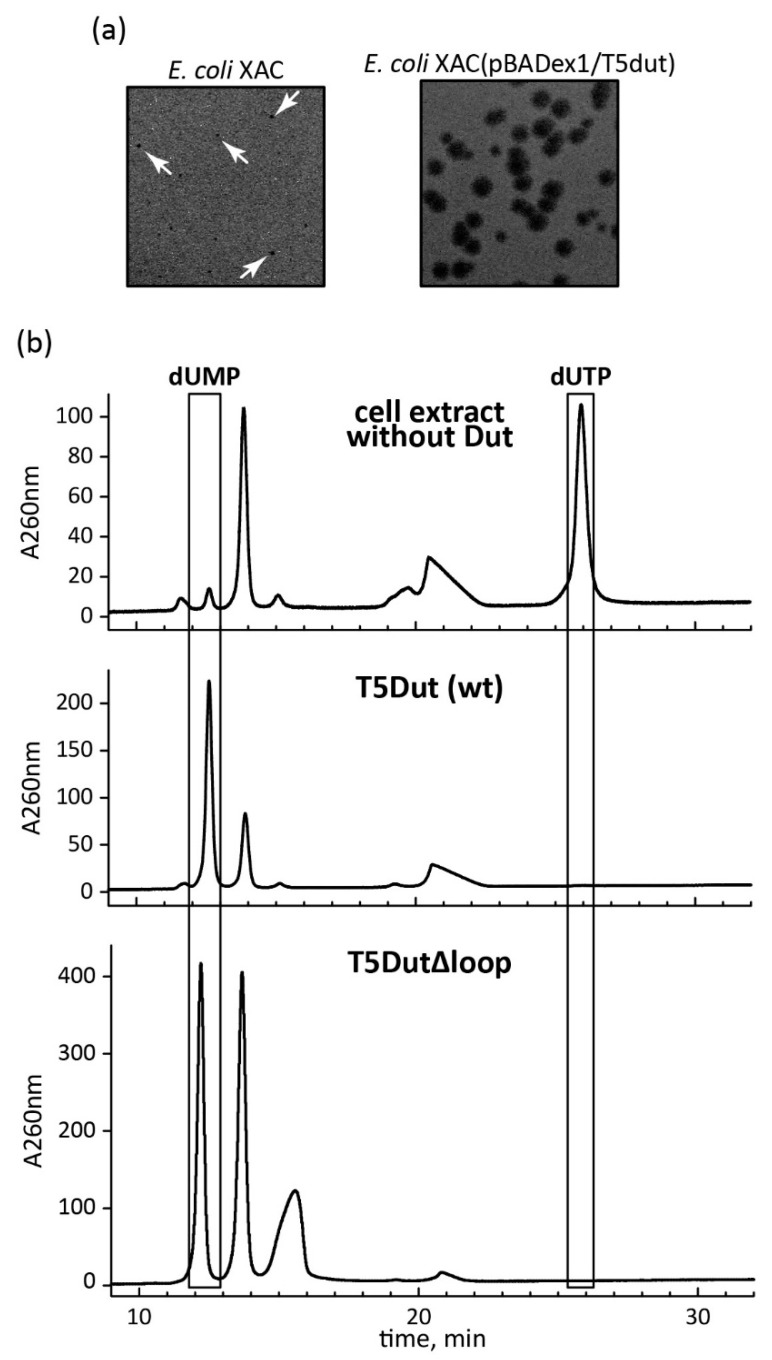
(**a**) Phenotypic manifestation of the loop reduction in the *dut* gene of phage T5. The mutant phage T5*dut*(Δloop) was plated at an equal titer onto a lawn of host *E. coli* strains XAC and the same cells carrying the plasmid for complementation pBADex1/T5dut (plaques are marked with arrows); (**b**) enzymatic activity of T5Dut and T5Dut(Δloop).

At the same time, the analysis of the amino acid sequence and the obtained structure allowed for the identification of an additional loop, a short polypeptide segment, six amino acid residues long (residues 30–35 in pdb 8QKY), located on the surface of each subunit of the trimer between β-strands β2 and β3 (Figure 2), which is presumably responsible for the new function of T5Dut. To test our hypothesis, the next mutant T5 phage T5*dut*(Δloop), with a dUTPase sequence 28-FFGTNPAADL-37 reduced to 28-FFNDL-32, was obtained. The mutant phage T5*dut*(Δloop) did not differ from the previously obtained mutant T5*dut*-am in terms of its main growth characteristics. To confirm that the loop reduction retains overall folding, without global changes, we obtained the mutant form T5DutΔloop structure (2.1 Å) (Table 1). A comparison of the crystal structures of wild-type T5Dut and its mutant form revealed no significant changes in the overall folding of the protein, except for in the mutated region. The superimposition of the wild-type and mutant forms, with an RMSD of 0.31 Å for the Cα atoms, shows the nearly identical folding of these proteins, with the only difference being observed in the region of interest (Figure 3). Moreover, this change in the amino acid sequence of T5 phage Dut had no impact on its enzymatic activity, specifically the hydrolysis of dUTP, as shown in the curve (Figure 4). The results of these experiments clearly demonstrate that the short segment of the polypeptide chain, consisting of six amino acid residues (30-GTNPAA-35), is responsible for carrying out an additional function of T5Dut.

### 2.3. The Canonical Function of T5 Phage dUTPase Is Not Crucial for Performing the Additional Function

In the study of the mutant T5 phage lacking phage-specific dUTPase activity during the development in *E. coli* cells, unfortunately, no mapping or description of the mutation had been performed [14].

**Figure 5:**
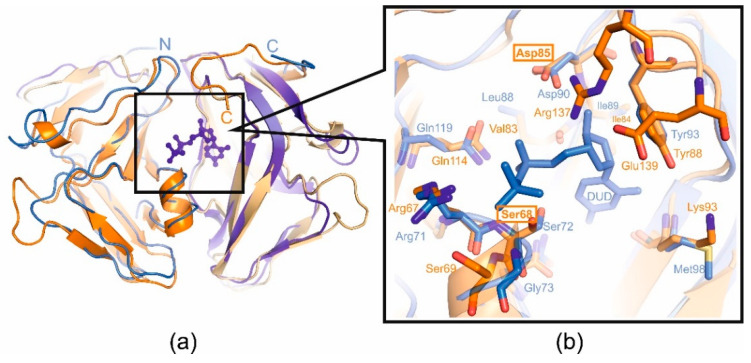
(**a**) Superimposed structural view of the dUTPases from *E. coli* in complex with the substrate analog deoxyuridine-5′-diphosphate (monomers are colored shades of blue, pdb id 1DUD) and T5 (monomers are colored in shades of orange, pdb id 8QKY); (**b**) active-site close-up showing the catalytically important residues. The mutated residues of the active center (Ser68 and Asp85) are boxed.

**Figure 6:**
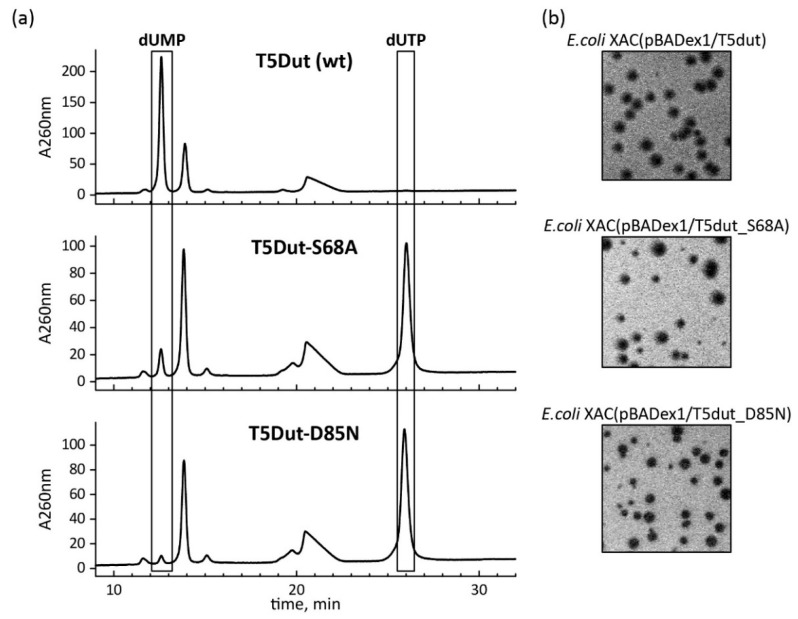
(**a**) Analysis of the enzymatic activity of T5Dut mutant forms T5Dut-S68A and T5Dut-D85N and (**b**) the gene’s ability to complement amber mutation in the T5 phage *dut* gene.

In order to determine whether enzymatic activity plays a role in the additional function of T5 phage dUTPase, the mutant forms of T5Dut that do not possess enzymatic activity were obtained. A comparison of the amino acid sequence and structure of the T5Dut phage with other characterized enzymes allowed us to identify two conserved amino acid residues (Ser68 and Asp85) (Figure 2a) within the active site that are directly involved in the enzymatic reaction in T5Dut (Figure 5). To terminate dUTPase activity, the plasmids encoding mutant forms of T5 phage dUTPase containing single amino acid substitutions, T5Dut-S68A and T5Dut-D85N, were obtained. These amino acid substitutions resulted in the complete loss of the enzymatic activity; however, no impact on the execution of the additional function of phage dUTPase was noted (Figure 6). These amino acid substitutions led to a complete loss of enzymatic activity (Figure 6a). In spite of this, the genes coding the mutant T5Dut-S68A and T5Dut-D85N efficiently complemented both the amber mutation in the *dut* gene T5*dut*-am (Figure 6b) and the mutation (deletion) in the T5*dut*(Δloop), demonstrating normal plaques.

All mutations pull, and their impacts on enzymatic activity and virus development are summarized in Table 3. Based on the results, we demonstrate that the enzymatic activity of the phage enzyme itself does not have a vital impact on the development of the phage. This indicates that the enzymatic activity and the newly detected function, combined within a single enzyme, are independent of each other.

## 3. Discussion

dUTPases participate in the regulation of the balance between dUTP and dTTP in cells and as a result, have a significant impact on the DNA repair mechanism in living organisms. Despite the fact that mutant organisms encoding inactive enzymes remain viable, a complete knockout of the *dut* gene has lethal consequences [3,4]. Thus, it is believed that enzymes of this class perform an additional function in the cell’s life processes that is independent of their enzymatic activity.

**Figure 7:**
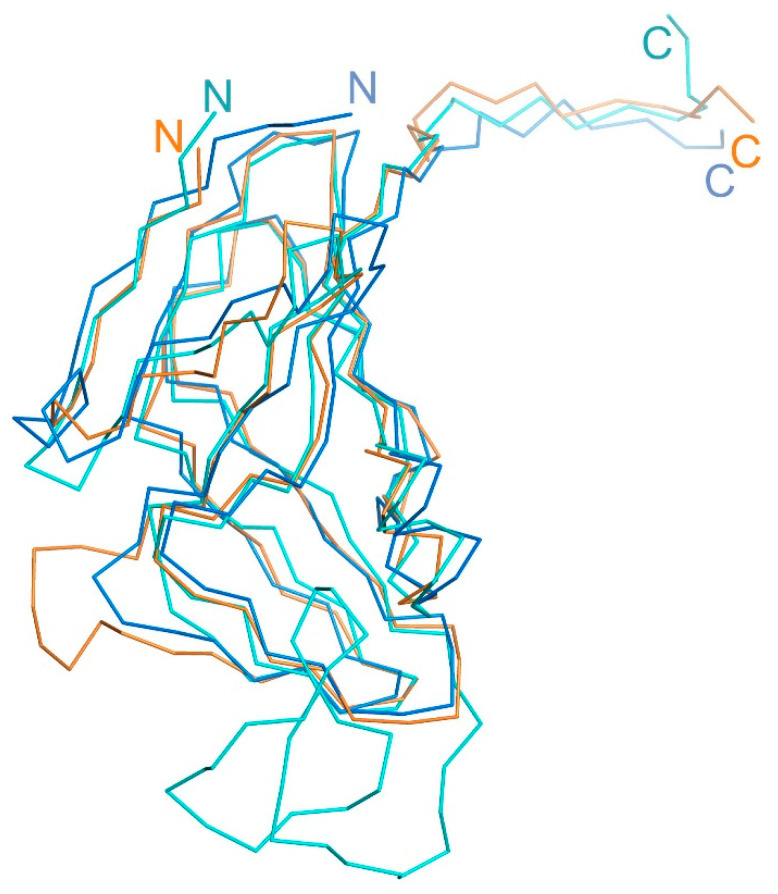
Superimposed structural view of the T5Dut (orange, pdb id 8QKY), *E. coli* (blue, pdb id 1DUD), and *S. aureus* (cyan, pdb id 4GV8) dUTPases monomers.

In this study, we demonstrate for the first time that the homotrimeric dUTPase of bacteriophage T5 performs an additional, non-enzymatic function in the virus’s life cycle. Thus, the Dut protein of phage T5 could belong to the narrow group of dUTPases confirmed to possess dual functions. For example, the involvement of dUTPase from several staphylococcal bacteriophages (ϕ11, 80α, ϕNM1, etc.) in the process of the derepression of pathogenicity islands (SaPI) during the infection of *Staphylococcus aureus* cells by the phage has been demonstrated [16]. The additional structural element on the surface of the trimeric (ϕ11, 80α) or dimeric (ϕNM1) forms of staphylococcal phage Dut plays a key role in carrying out this function. This element is formed by an extended polypeptide chain of up to 40 amino acid residues in length (Figure 7). The next example is the dUTPase of *M. smegmatis*, which possess a short specific loop in the C-terminus, contributing to an additional protein function. Our experimental results clearly demonstrate that, in the case of T5Dut, the presence of an additional function is also attributed to the presence of an extra element in the enzyme structure (a short loop consisting of only six amino acids (30-GTNPAA-35)). While its reduction does not impact the overall spatial organization of the enzyme nor its ability to cleave the substrate (dUTP), it has severe consequences for the development of the T5 bacteriophage itself. The fact that the additional function of T5Dut is likely realized at some stage of the phage’s development significantly distinguishes it from the dUTPases of the bacteria and staphylococcal phages. However, similar to the previously mentioned examples of dUTPases in the staphylococcal phages, *E. coli*, *M. smegmatis*, and potentially other organisms, the additional function of T5Dut is independent of the enzymatic activity of the protein.

The details of the process in which this additional function of the T5Dut phage is realized are yet to be uncovered. However, based on the results of our additional experiments, it becomes evident that this enzyme plays a direct role in the phage particle morphogenesis process, and the new function is specifically realized at this stage of virus development in *E. coli* cells. The fact that the Dut gene of the T5 bacteriophage is necessary for the late stages of viral development partially explains the phenotypic manifestation of the mutation in the *dut* gene, which results in an approximately tenfold decrease in phage yield. It is possible that the phage possesses some “bypass pathways” to complete the formation of viral particles, despite such significant losses in yield. At the same time, we cannot dismiss the possibility that the absence of dUTPase in certain T5-like phages (Figure S1) may lead to more dramatic consequences, despite their amino acid sequences being practically identical.

## 4. Materials and Methods

### 4.1. Construction of Plasmid

DNA cloning was performed using standard methods and techniques [17]. Details of the plasmid construction are described in the Supplementary Materials.

The plasmid pZ/T5dut-am contained a fragment of the *D14* and *dut* gene region of the T5 phage, with an amber mutation in the *dut* gene (the codon of Ser69 was substituted for an amber codon). The plasmid pZ/T5dutΔloop contained a fragment of the *D14* and *dut* gene region of the T5 phage, with short deletions in the *dut* gene (88–93 and 96–104 bp). These plasmids were used to generate the corresponding mutants of the T5 phage.

The plasmid pBADex1/T5dut contains the wild-type *dut* gene under the control of an arabinose promoter. The plasmid pBADex1/T5dutΔloop encodes a mutant form of the T5 phage Dut, in which the amino acid sequence 28-FFGTNPAADL-37 is replaced with 28-FFNDL-32. Plasmids pBADex1/T5dut_S68A and pBADex1/T5dut_D85N encode mutant forms of the T5 phage dUTPase, with the corresponding amino acid substitutions that result in the loss of enzymatic activity. These described plasmids were used for the protein production and complementation tests.

### 4.2. Bacteriophages and Bacterial Strains

Strain *E. coli* TOP10 (F^−^, *mcr*A, Δ(*mrr*-*hsd*RMS-*mcr*BC), ϕ80*lac*ZΔM15, Δ*lac*X74, *deo*R, *rec*A1, *ara*D139, Δ(*ara-leu*)7697, *gal*U, *gal*K, *rps*L (str^R^), *end*A1, *nup*G) was used as the host for plasmid construction and propagation. Strain *E. coli* XA101 (*ara*, Δ(*lac*-*pro*), *sup*D, *gyr*A, *met*B, *arg*E-am, *rpo*B, *thi*) was used to cross phage T5 and grow its amber mutant, T5*dut*-am. The non-permissive *E. coli* XAC strain (*ara*, Δ(*lac-pro*), *gyr*A, *arg*E-am, *rpo*B, *thi*) was employed for the selection of T5 phage mutants carrying an amber mutation in the *dut* gene. The expression of both the wild-type T5 phage *dut* gene and its mutant variants was carried out in the *E. coli* XAC strain.

Bacteriophage T5*dut*-am, carrying an amber mutation in the *dut* gene, was generated through the recombination of phage T5 with the pZ/T5dut-am plasmid, as described in the Supplementary Materials. Bacteriophage T5*dut*(Δloop) encodes a mutant form of dUTPase, in which five amino acid residues (sequence 28-FFGTNPAADL-37) have been deleted and replaced with 28-FFNDL-32. It was obtained through the recombination of phage T5 with the pZ/T5dutΔloop plasmid.

### 4.3. Expression and Purification of Proteins

The *E. coli* XAC strain transformed with the respective plasmids was used for the expression of the wild-type *dut* gene and its mutant form, *dut*Δloop. The cells were grown in LB medium supplemented with Amp (100 µg/mL) at +37 °C until reaching an OD600 of 0.5. Gene expression was induced by adding L-(+)-Arabinose (Sigma-Aldrich, Taufkirchen, Germany) to a final concentration of 0.2% (*w*/*v*), and the cells were further incubated at +37 °C for 4 h. The cells were pelleted via centrifugation at 6000× *g* for 20 min. The cell pellet was then resuspended in lysis buffer (20 mM Tris HCl pH 8.0, 50 mM NaCl) and disrupted using a Gaulin Homogenizer (APV Homogenizer GmbH). The insoluble fraction was separated via centrifugation at 30,000× *g* for 30 min. The soluble fraction was used for the isolation of the T5Dut and the mutant form T5DutΔloop.

The cell extract was supplemented with streptomycin sulfate to a concentration of 2% (*w*/*v*), incubated for 1 h at +4 °C, and centrifuged for 30 min at 20,000× *g*. The supernatant was diluted tenfold with buffer A (20 mM Tris HCl pH 8.5, 1 mM MgCl_2_), filtered through a 0.22 μm filter (Millipore, Billerica, MA, USA), and applied to a Mono Q 10/100 GL column (GE Healthcare, Chicago, IL, USA) equilibrated with buffer A. Elution was carried out at a flow rate of 2 mL/min, with a linear gradient of 0–0.3 M NaCl, in buffer A for 50 min. Fractions containing T5Dut (T5DutΔloop) according to SDS PAGE were combined, diluted threefold with buffer B (20 mM Na-Acetate, pH 5.0), supplemented with glacial acetic acid to a concentration of 6 mM, and applied to a Mono S 5/50 GL column (GE Healthcare, Chicago, IL, USA) in buffer B. Elution was carried out at a speed of 0.6 mL/min with a NaCl concentration gradient in buffer B: from 0 to 100 mM in 5 min and from 100 to 300 mM in 45 min. Fractions containing T5Dut (T5DutΔloop) according to SDS PAGE were pooled, concentrated using an Amicon centrifugal concentrator MWCO 10 kDa (Millipore Ireland Ltd., Tullagreen, County Cork Carrigtwohill, Ireland), and applied to a Superdex 200 10/300 GL (GE Healthcare, Chicago, IL, USA) gel filtration column in buffer C (20 mM Tris HCl, pH 8.0, 150 mM NaCl).

### 4.4. dUTPase Activity Test

The expression of the wild-type T5 *dut* gene and its mutant forms (*dut-S68A*, *dut-D85N*, and *dut*(Δloop)) was conducted as mentioned above. A 0.5 mL suspension of cells with an OD600 of 0.5 was sonicated, and the soluble fraction was used to assess enzymatic activity. The *E. coli* XAC strain transformed with the pBADex1 plasmid was used as a negative control.

Then, 60 μL of buffer—20 mM Tris HCl, pH 8.0, 100 mM NaCl, 5 mM MgCl_2_ containing 200 mkM dUTP—was supplemented with 0.25 μL of cell extract. The mixture was incubated for 30 min at +35 °C, and then 180 μL of 20 mM ammonium acetate, pH 8.0, 10 mM EDTA was added. A 200 μL aliquot of the sample was immediately applied to a Mono Q 5/50 GL column (GE Healthcare, Chicago, IL, USA) equilibrated with 20 mM ammonium acetate, pH 8.0 buffer. Elution was carried out at flow rate of 0.6 mL/min for 30 min by linearly increasing the ammonium acetate concentration up to 1.2 M.

Registration was conducted through absorption at 260 nm.

### 4.5. Crystallization and Crystallography

Screening for the initial crystallization conditions was performed using the sitting drop vapor diffusion method with commercial screen sets (Hampton Research, Aliso Viejo, CA, USA). Here, 0.2 µL of protein (10–15 mg/mL) was combined with 0.2 µL of reservoir solution and incubated at +23 °C. Crystals of T5Dut were obtained by drop equilibrating against 0.075 M Tris HCl, pH 8.5, 1.5 M ammonium sulfate, and 25% (*v*/*v*) glycerol (A4 from Crystal Screen Cryo, Hampton Research, Aliso Viejo, CA, USA), which appeared overnight and reached their full size within 3 days. T5DutΔloop crystals were obtained with 0.085 M HEPES sodium, pH 7.5, 1.7% (*v*/*v*) polyethylene glycol 400, 1.7 M ammonium sulfate, and 15% (*v*/*v*) glycerol (D3 Crystal Screen Cryo, Hampton Research, Aliso Viejo, CA, USA) in the same manner. The crystals were harvested and flash-frozen.

Diffraction data from the T5Dut crystal was collected on a beamline P11 at PETRA III, DESY, Hamburg, Germany. The reflection dataset was processed using XDS [18]. The diffraction data from the T5DutΔloop were collected using a home-source Rigaku XtaLAB Synergy-S laboratory system (The Woodlands, TX, USA) [19]. The reflection data were processed and merged using CrysAlis software 42.89a [20]. The structures were determined using molecular replacement with Phaser [21], with the structure of dUTPase from *E. coli*, determined at a 1.9 Å resolution (pdb id 1DUP), being used as a search model. The water molecules and metal ions were removed from the model. The initial model was subjected to crystallographic refinement with REFMAC5 [22,23]. The manual rebuilding of the model was carried out in Coot [24]. The final cycle, with occupancy refinement, was performed in Phenix [25]. The data and refinement statistics are summarized in Table 2. The atom coordinates and structure factors were deposited in the Protein Data Bank. Figures were prepared using PyMOL [26].

## 5. Conclusions

We have identified a structural element within T5Dut, a small-loop 30-GTNPAA-35 that is only six amino acids long, which is responsible for the implementation of a novel function. Reducing the size of this loop has dramatic consequences for the development of the phage T5i n *E. coli* cells, which are similar to those obtained with the complete removal of Dut. Additionally, we have demonstrated that the enzymatic activity is implemented separately and does not influence the novel function. We speculate that the novel function is realized during the morphogenesis stage of the phage particle.

## Figures and Tables

**Table 1:** Efficiency of plating T5wt and T5*dut*-am phages on various *E. coli* hosts.

*E. coli*	Efficiency of Plating Compared to That Using *E. coli* XA101	
Strain	T5wt	T5*dut*-am
XA101 (Su+)	1	1
XAC (Su0)	0.97	0.33

**Table 2:** Crystallographic data collection and refinement statistics.

Data Collection		
	T5Dut	T5DutΔloop
Wavelength (Å)	1.0332	1.5418
Resolution range (Å)	50.00–2.00 (2.05–2.00) ^a^	23.00–2.10 (2.20–2.10)
Space group	P4_3_	P6_5_
Cell parameters		
a, b, c (Å)	78.4, 78.4, 86.9	88.7, 88.7, 100.0
α, β, γ (◦)	90.0, 90.0, 90.0	90.0, 90.0, 120.0
Collection temperature (K)	100	120
Total reflections	452,788 (20,225)	233,256 (30,793)
Unique reflections	35,488 (2637)	25,912 (3239)
R_merge_ (%)	7.3 (101.5)	9.4 (51.4)
Multiplicity	12.76 (6.67)	9.00 (9.51)
Completeness (%)	99.9 (99.9)	99.3 (100.0)
Mean I/sigma(I)	22.91 (1.86)	28.14 (4.22)
Wilson B-factor (Å^2^)	35.1	23.8
CC_1/2_	0.99 (0.72)	0.99 (0.95)
Refinement		
Resolution range	26.76–2.00 (2.06–2.00)	20.00–2.10 (2.15–2.10)
Reflections used in refinement	35,476 (2598)	23,632 (1746)
Reflections used for R-free	1775 (137)	1160 (76)
R-work, %	17.76 (27.97)	22.05 (31.7)
R-free, %	21.09 (33.25)	26.41 (40.6)
RMSD bond lengths (Å)	0.007	0.007
RMSD bond angles (◦)	0.843	1.342
Ramachandran favored (%)	98.51	97.12
Ramachandran allowed (%)	1.49	2.88
Ramachandran outliers (%)	0.00	0.00
Average B-factor (Å^2^)	40.21	33.46
Macromolecules	40.24	33.48
Ligands	43.65	42.32
Solvent	39.63	30.51
PDB ID	8QKY	8QLD

^a^ Values in parentheses are for the highest resolution shell.

**Table 3:** Complementation tests for various T5 dUTPases on mutant T5 phages.

Strain	Phage T5*dut*-am			Phage T5*dut*(Δloop)		
	Enzymatic Activity	Additional Function	Phenotype Manifestation	Enzymatic Activity	Additional Function	Phenotype Manifestation
*E. coli* XAC (Su0)	−	−	defective	+	−	defective
*E. coli* XAC (pBADex1/T5dut)	+	+	wt	+	+	wt
*E. coli* XAC (pBADex1/T5dut_S68A)	−	+	wt	+	+	wt
*E. coli* XAC (pBADex1/T5dut_D85N)	−	+	wt	+	+	wt
*E. coli* XAC (pBADex1/T5dutΔloop)	+	−	defective	+	−	defective

## Data Availability

The data supporting the findings of this manuscript are available from the corresponding authors upon reasonable request. The coordinates of the T5Dut and T5DutΔloop studied by X-ray crystallography have been deposited in the PDB with the accession codes 8QKY and 8QLD, respectively.

## Supplementary Materials

The following supporting information can be downloaded at: https://www.mdpi.com/article/10.3390/ijms25020892/s1.
