# Supplementary material for: Bacteriophage T5 dUTPase: Combination of Common Enzymatic and Novel Functions
Source: Int J Mol Sci. 2024 Jan 10;25(2):892. doi: 10.3390/ijms25020892 (PMC10815766; doi:10.3390/ijms25020892)
Supplement: Supplementary file 1 [file ijms-25-00892-s001.zip › ijms-2750491-supplementary.pdf]

Supplementary Materials

Oligonucleotides:

#1: 5`-GCTGCTGTAGGTCAAGATGTGTTAGATAAG-3`;
#2: 5`-TGGCGCTTACCCAGCTAGCTACGAGGAAC-3`;
#3: 5`-GTTCCTCGTAGCTAGCTGGGTAAGCGCCA-3`;
#4: 5`-CTTCCAAATCCGCCTTCTCCAC-3`;
#5: 5`-CGTAAATCATTAAAGAATGCTCGCAGATCCA-3`;
#6: 5`-CGAGCATTCTTTAATGATTTACGAGCTATTGCACCAG-3`;
#7: 5`-GCGTGCATATGATTAAAATTAAGTTAACTC-3`;
#8: 5`-ACTGTCGACTTATTTGCTTCCTGAAC-3`;
#9: 5`-CTTACCTAAAGACGCACGAGGAACCAC-3`;
#10: 5`-GTGGTTCCTCGTGCGTCTTTAGGTAAG-3`;
#11: 5`-CGATAGTCTGAGTTGATCACTCCTGC-3`;
#12: 5`-GCAGGAGTGATCAACTCAGACTATCG-3`.

Construction of Plasmid

DNA fragments containing the amber mutation in the *dut* gene were obtained through PCR-based oligonucleotide-directed mutagenesis. DNA fragments, 283 bp and 239 bp in length, which were amplified using two pairs of primers (#1 and #2, and #3 and #4, respectively), were mixed in equimolar amounts and used as a template for amplifying a fragment with primers #1 and #4. The resulting 493 bp fragment was cloned into the *Eco*RV site of the pZErOR-2 vector ("Invitrogen").

The plasmid pZ/T5dut(Δloop), containing a DNA fragment with short deletions in the *dut* gene, was obtained using a similar method. For this, DNA fragments of 160 bp and 341 bp, amplified with two pairs of primers (#1 and #5, and #6 and #4, respectively), were mixed in equimolar amounts and used as a template for amplifying a fragment with primers #1 and #4. The resulting 478 bp fragment was cloned into the *Eco*RV site of the pZErOR-2 vector.

The plasmid pBADex1/T5dut carries the wild-type *dut* gene of T5 phage under the control of an arabinose promoter. It was obtained by cloning a 449 bp DNA fragment, amplified with primers #7 and #8, into the *Nde*I and *Sal*I sites of the pBADex1 vector (derived from the pBAD18 vector; kindly provided of Kaliman A.V., Institute of Protein Research RAS, Pushchino, Russia).

The plasmid pBADex1/T5dut_S68A encodes a mutant form of T5 phage Dut with an amino acid substitution that leads to the loss of dUTPase activity. The DNA fragment containing the mutation in the *dut* gene was obtained using PCR-based oligonucleotide-directed mutagenesis. For this, DNA fragments of 224 bp and 267 bp, amplified with two pairs of primers (#7 and #9, and #8 and #10, respectively), were mixed in equimolar amounts and used as a template for amplifying a fragment with primers #7 and #8. The resulting 449 bp fragment was cloned into the *Nde*I and *Sal*I sites of the pBADex1 vector.

The plasmid pBADex1/T5dut_D85N, which encodes an inactive mutant form of T5 phage Dut, was obtained using a similar method. For this, DNA fragments of 274 bp and 216 bp, amplified with two pairs of primers (#7 and #11, and #8 and #12, respectively), were mixed in equimolar amounts and used as a template for amplifying a fragment with primers #7 and #8. The resulting 449 bp fragment was cloned into the *Nde*I and *Sal*I sites of the pBADex1 vector.

Construction of T5dut-am and T5dut(Δloop) mutants

The *E.coli* XA101 strain cells, transformed with either pZ/T5dut-am or pZ/T5dut(Δloop) plasmids, were grown at +37°C in LB medium supplemented with Kan (40 μg/mL) until reaching an OD600 of 0.5. Then, 1 mL of the culture was infected with T5wt phage at a multiplicity of infection (MOI) of 10. After 10 minutes of phage adsorption at room temperature, the cells were pelleted by centrifugation at 6,000g for 1 minute. The cell pellet was resuspended in 1 mL of fresh LB medium, and the infected cells were incubated at +37°C for 90 minutes until complete lysis of the culture occurred.

Debris was pelleted at 6,000g for 5 minutes and phage progeny were spread using the double-layer agar method onto corresponding strains of *E.coli*. For T5*dut*-am phage, a permissive strain of *E.coli* XA101 was used as the host. For T5*dut*(Δloop) phage, the *E.coli* XAC strain transformed with the pBADex1/T5dut plasmid was used as the host. The agar medium contained 100 μg/mL ampicillin and 0.1% (w/v) L-(+)-arabinose. Isolated phage colonies were transferred onto two strains of *E.coli*, XA101 and XAC, in the case of selecting T5*dut*-am phage, and XAC strain transformed with the pBADex1/T5dut plasmid, and XAC strain in the case of selecting T5*dut*(Δloop) phage. Negative colonies that exhibited growth differences on the respective strains were selected and verified by sequencing.

```
                                              Motif I
Dut_E.coli          MKKIDVKILDPRVGKEFPLPTYATSGSAGLDLRACLN-----DAVELAPGDTT
Dut_phi3            MQPIRLVVAN-----ENCKPHVGSAEAAGMDLRMNVKT--ATGFTPFLPNQVL
Dut_pVP-1           MEKIRIKLLN-----EKAEPRCMKDGDAGIDLRMNIET--AQGFTPLLRGESI
Dut_My1             ---MEIKLAY-----LECMPHVGSEEAAGMDLRLYLGNKSTDIMTVIPPGETK
Dut_phiR2-01        --MLKIKLSH-----PDCMPKIGTEYSAGMDLKAFFGTNQNSDLRAIQPGKDL
Dut_Eps7            --MIKIKLTH-----PDCMPKIGSDDAAGMDLRAFFGTNLAADLRAIAPGKSL
Dut_H8              --MIKIKLTH-----PDCMPKIGSNDAAGMDLRAFFGTNLAADLRAIAPGKSL
Dut_SPC35           --MIKIKLTH-----PDCMPKIGSEDAAGMDLRAFFGTNPAADLRAIAPGKSL
Dut_slur09          --MIKIKLTH-----PDCMPKIGSEDAAGMDLRAFFGTNPAADLRAIAPGKSL
Dut_vBEcoSAKFV33    --MIKIKLTH-----PDCMPKIGSEDAAGMDLRAFFGTNPAADLRAIAPGKSL
Dut_phi5            --MIKIKLTH-----PDCMPKIGSEDAAGMDLRAFFGTNPAADLRAIAPGKSL
Dut_T5              --MIKIKLTH-----PDCMPKIGSEDAAGMDLRAFFGTNPAADLRAIAPGKSL
Dut_DT57C           --MIKIKLTH-----PDCMPKIGSEDAAGMDLRAFFGTNPAADLRAIAPGKSL
                       : : :          *    .   **:**:   .            :  ..

                                 Motif II            Motif III
Dut_E.coli          LVPTGLAIHIADPSLAAMMLPRSGLGHKHGIVLGNLVGLIDSDYQGQLMISVW
Dut_phi3            KFGTGVKIEIPKG-WVGLVMPRSGLGTKYEVTLLNTVGVIDSDYRGEIQVAIR
Dut_pVP-1           TFGTGVKMAIPRG-WVGLIMPRSGLGFKYEIRLANTTGVIDSNYRGEIMVKMR
Dut_My1             RYRTGVSVAIPKG-WVGLIAPRSSTG-RLKCRLANTLGVIDSDYRGELAMEIT
Dut_phiR2-01        MIDTGVAMSIPEG-WCGFVLPRSSTG-KLHCKLANTVGLIDSDYTGNIKLLVH
Dut_Eps7            MIDTGVAVEIPRG-WFGLVVPRSSLG-KRKLMIANTAGVIDSDYRGTIKMNLY
Dut_H8              MIDTGVAVEIPRG-WFGLVVPRSSLG-KRKLMIANTAGVIDSDYRGTIKMNLF
Dut_SPC35           MIDTGVAVEIPRG-WFGLVVPRSSLG-KRHLMIANTTGVIDSDYRGTIKMNLF
Dut_slur09          MIDTGVAVEIPRG-WFGLVVPRSSLG-KRHLMIANTAGVIDSDYRGTIKMNLF
Dut_vBEcoSAKFV33    MIDTGVAVEIPRG-WFGLVVPRSSLG-KRHLMIANTAGVIDSDYRGTIKMNLF
Dut_phi5            MIDTGVAVEIPRG-WFGLVVPRSSLG-KRHLMIANTAGVIDSDYRGTIKMNLF
Dut_T5              MIDTGVAVEIPRG-WFGLVVPRSSLG-KRHLMIANTAGVIDSDYRGTIKMNLY
Dut_DT57C           MIDTGVAVEIPRG-WFGLVVPRSSLG-KRHLMIANTAGVIDSDYRGTIKMNLY
                       **: : *      .:: ***. * :    : *  *:***:* * : : :

                             Motif IV                    Motif V
Dut_E.coli          NRGQDSFTIQPGERIAQMIFVPVVQ--AEFNLVEDFDATDRGEGGFGHSGRQ
Dut_phi3            NRGDKEVMLEDYERVCQMVLVPHYLVFNNLEYVDSLSETERGENGHGSSGKL
Dut_pVP-1           NCGDEDVFLEDFERVCQMVIVPHYVVHNNLEFVDELDETNRGESGFGESGRQ
Dut_My1             NDGTEDVILENFQRIVQMVIVPHYNPH-SFTVVDELSETNRGEKGWGSSGKV
Dut_phiR2-01        NYGKEIVTLENFQRLCQIVVVPHYPVH-NFEIVDSLEETDRGTGGFGSTGSK
Dut_Eps7            NYGSEMQTLENFERLCQLVVLPHYSTH-HFEIVDELEETDRGEGGFGSSGSK
Dut_H8              NYGSEMQTLENFERLCQLVILPYYSTH-HFEIVDELEETDRGEGGFGSSGSK
Dut_SPC35           NYGSEIQTLENFERLCQLVILPHYSTH-NFEIVDELGETNRGEGGFGSSGSK
Dut_slur09          NYGSEIQTLENFERLCQLVVVPHYSTH-NFEIVDKLGETNRGEGGFGSSGSK
Dut_vBEcoSAKFV33    NYGSEIQTLENFERLCQLVVVPHYSTH-NFEIVDELGETNRGEGGFGSSGSK
Dut_phi5            NYGSEIQTLENFERLCQLVVLPHYSTH-HFEIVDELEETDRGEGGFGSSGSK
Dut_T5              NYGSEMQTLENFERLCQLVVLPHYSTH-NFKIVDELEETIRGEGGFGSSGSK
Dut_DT57C           NYGSEIQTLENFERLCQLVVLPHYSTH-NFKIVDKLEETDRGEGGFGSSGSK
                    * * .    ::  :*: *::.:*         :  *:.:  * **  * * :*
```

Figure S1. Sequence alignment of trimeric dUTPases; phi 3,Vibrio phage [YP_009207587.1]; pVp-1, Vibrio phage [YP_007007825.1]; My1, Pectobacterium phage [YP_006906377.1]; phiR2-01, Yersinia phage [YP_007237100.1]; Eps7, Escherichia phage [YP_001837067.1]; H8, Escherichia phage [YP_009966079.1]; SPC35, Salmonella phage [YP_004306604.1]; slur09, Escherichia phage [YP_009202116.1]; vB_EcoS_AKFV33, Escherichia phage [YP_006382432.1]; phi5, Salmonella phage; DT57C, Escherichia phage [YP_009149887.1]. Extra loop are boxed
